# The brain‐before‐heart strategy for coronary artery bypass grafting in the severely atherosclerotic aorta: A single‐institution experience

**DOI:** 10.1002/clc.23913

**Published:** 2022-09-19

**Authors:** Rakan I. Nazer, Ali M. Albarrati

**Affiliations:** ^1^ Department of Cardiac Science, King Fahad Cardiac Center, College of Medicine King Saud University Riyadh Kingdom of Saudi Arabia; ^2^ Department of Rehabilitation Science, College of Applied Medical Science King Saud University Riyadh Kingdom of Saudi Arabia

**Keywords:** ascending aorta, atherosclerosis, revascularization, stroke

## Abstract

**Background:**

Severe atherosclerosis of the ascending aorta (SAA) in patients undergoing surgical revascularization by coronary artery bypass grafting (CABG) is becoming an increasing problem as more elderly patients are diagnosed with coronary artery disease. Strokes and other neurologic insults are common complications in this group, with devastating impacts on outcomes and prognoses.

**Hypothesis:**

Early detection of the atherosclerotic aorta and the application of a stroke prevention protocol will reduce the risk of stroke in patients with SAA.

**Methods:**

In 2012, we adopted a protocol devised to preemptively detect and manage patients suspected of having SAA. From the time of the application of the protocol, we compared the immediate and late outcomes of CABG in SAA in the 8 years preceding the protocol in a “control” group (30 patients) and in the 8 years following the protocol in a “brain” group (69 patients).

**Results:**

More patients with SAA were detected after the initiation of the protocol. They had significantly more history of stroke, renal dysfunction, and left main coronary disease. The percutaneous coronary intervention was utilized more after the protocol (26% vs. 7%) and there was far less utilization of replacement of the ascending aorta (12% vs. 37%). Postoperative stroke rates were significantly less after the protocol (2% vs. 18%), with an almost twofold reduction in stroke associated with SAA even after risk adjustment. The composite endpoints of cardiac death, nonfatal myocardial infarction, and stroke were significantly reduced after initiating the protocol at a median of 2.3 years from the time of revascularization.

**Conclusion:**

Early detection of SAA and individualized therapeutic strategies for revascularization is effective in reducing athero‐embolic brain injury and are associated with better prognosis.

## 1 INTRODUCTION

Severe ascending aortic atherosclerosis (SAA) has been shown to be the most important predictor of stroke in coronary artery bypass grafting (CABG).^1^ The increasing number of elderly patients undergoing surgical revascularization in recent years has brought greater attention to SAA in CABG, as atherosclerotic disease increases with age.^2^ The underlying potential risk for athero‐embolic events is closely correlated with the severity of atherosclerosis in the ascending aorta and adds a considerable risk of spontaneous embolic strokes with diminished survival during the long‐term postoperative course.^3^ Surgical manipulation of the ascending aorta during cardiac surgery procedures, and in particular the application and removal of the aortic cross‐clamp, is associated with the highest number of detectable emboli in the middle cerebral artery by Doppler ultrasound.^4^ Increasing awareness of SAA is the first crucial step in limiting and preventing its devastating complications.^5^

Increasing awareness starts with a multitude of gradual steps that include taking a proper history, performing focused preoperative imaging, and careful intraoperative assessment of atheroma by scanning the ascending aorta. The revascularization strategy and planned surgical techniques will need to be custom‐tailored to the individual. This includes opting for percutaneous coronary intervention (PCI) rather than CABG, hybrid revascularization (PCI + CABG), performing CABG without cardiopulmonary bypass (CPB) support (off‐pump coronary artery bypass [OPCAB]), performing CABG under CPB support and fibrillatory arrest with intermittent reductions or interruptions in CPB flow to construct proximal anastomoses (fibrillatory arrest coronary artery bypass [FACAB]), and performing CABG after graft replacement of the atherosclerotic aorta (aorta replacement coronary artery bypass [ARCAB]). The purpose of this study was to conduct a retrospective cohort review of the immediate and late outcomes of isolated CABG in patients with SAA before and after the implementation of the preemptive diagnosis and management measures for SAA.

## 2 METHODS

**Figure 1:**
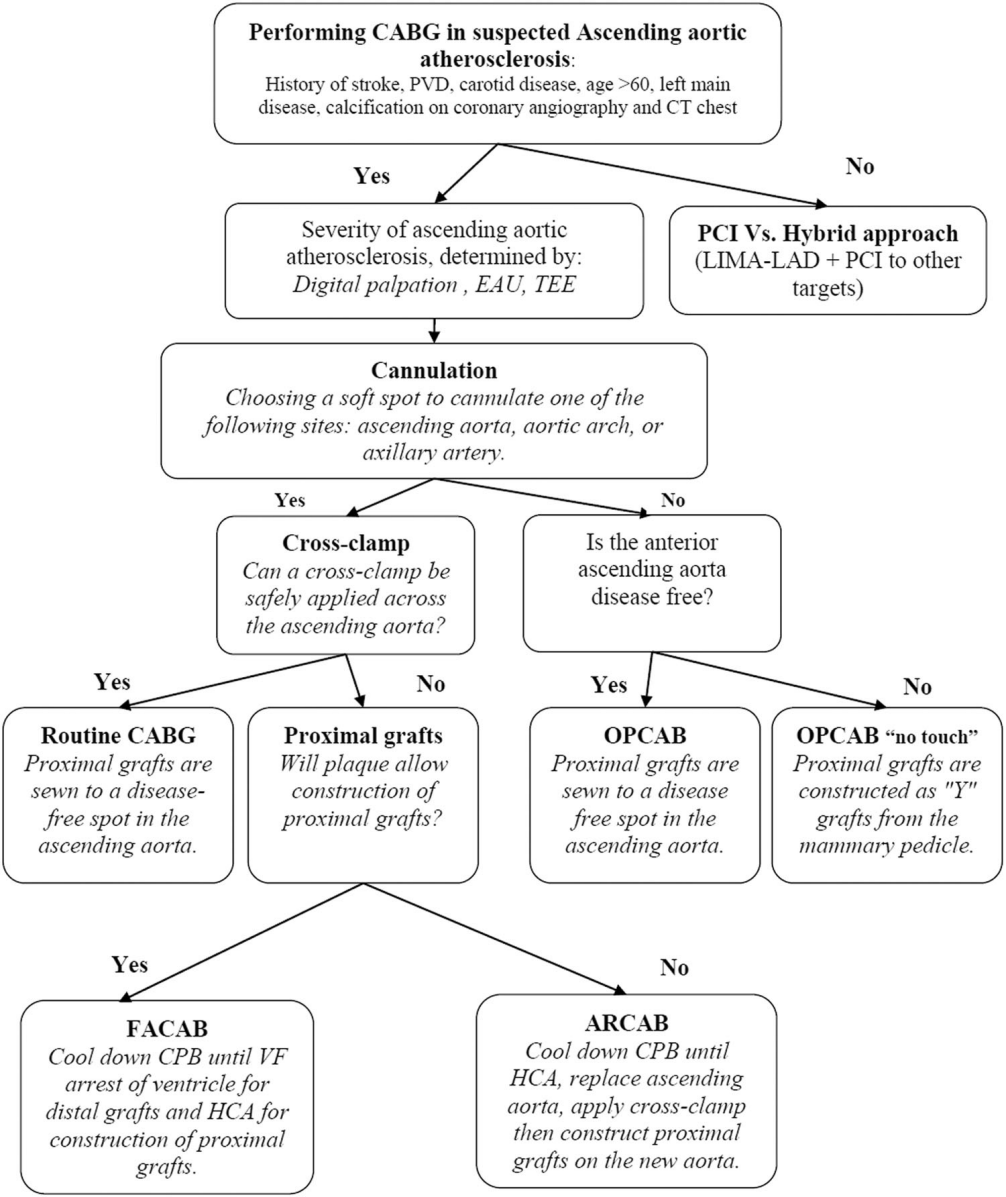
“Brain‐before‐heart” protocol for coronary bypass surgery in severe ascending aorta atherosclerosis. ACAB, fibrillatory arrest coronary artery bypass; ARCAB, aorta replacement coronary artery bypass; CPB, cardiopulmonary bypass; CT, computerized tomography; EAU, epiaortic ultrasound; FHCA, hypothermic circulatory arrest; LAD, left anterior descending; LIMA, left internal mammary; OPCAB, off‐pump coronary artery bypass; PCI, percutaneous coronary intervention; PVD, peripheral vascular disease; TEE, trans‐esophageal echocardiography.

In an effort to reduce the incidence of stroke post‐CABG in patients with SAA, our local heart team initiated a stroke prevention protocol starting in July 2012. This was conveniently labeled the “brain‐before‐heart” revascularization strategy. The strategy is predicated on increasing awareness of ascending aortic atherosclerosis to prevent the devastating complication of perioperative stroke. This was conducted through a stepwise process involving an initial risk stratification, preoperative noninvasive imaging, individualized revascularization planning, intraoperative assessment of the ascending aorta, and the use of alternative surgical techniques guided by the site and burden of atheroma (Figure 1).

A high index of suspicion is given to patients who present with any of the following: a history of stroke, peripheral vascular disease, carotid artery disease, left main stenosis, and/or heavy calcification on coronary angiography. For preoperative imaging, patients who are >60 years of age undergo a plain computed tomography (CT) scan of the chest to detect signs of calcification in the thoracic aorta. Carotid doppler ultrasound (US) is done routinely before CABG.

The extent and burden of atheroma in the ascending aorta are subjected to careful assessment and preoperative planning by the heart team to decide whether to pursue surgical revascularization, PCI, or a hybrid approach. All patients undergoing CABG are subjected intraoperatively to epiaortic US (EAU) scanning and careful digital palpation of the ascending aorta to choose a soft spot for cannulation. Depending on the site and burden of atheroma in the ascending aorta, the surgical team can tailor an individualized surgical strategy involving any of the following surgical techniques: routine CABG with cross‐clamp, OPCAB, FACAB, or ARCAB.^5^, ^6^, ^7^, ^8^, ^9^ After the approval of our local institutional ethics board, a systematic retrospective chart review was conducted according to the guidelines of the Declaration of Helsinki on all adult patients who were referred for isolated surgical revascularization at our local institution between 2004 and 2019.

Patient‐specific information was censored and individual consent was waived using the time of the implementation of the brain‐before‐heart stroke prevention protocol as a pivotal set‐point (July 2012). The selected cohort of patients with SAA was divided into a control group C, comprising patients referred for CABG from January 2004 to June 2012, and a study group labeled the brain group B, comprising patients referred for CABG from July 2012 to December 2019. All surgical revascularization was performed via a median sternotomy with bypass conduits selected according to the preference of the operating team. These included at least one left internal mammary artery (LIMA) and/or a combination of saphenous vein grafts (SVG), right internal mammary artery (RIMA), and radial artery (RA).

EAU scanning of the ascending aorta was adopted only after the implementation of the stroke prevention protocol. Before this, the assessment of atheroma in the ascending aorta was done by digital palpation. Our protocol is as follows: Depending on the severity of ascending aorta disease and the site of the atheroma, a decision is made to choose between using OPCAP (with or without “no touch”) or utilizing CPB via the heart–lung machine. Aortic “no touch” revascularization strategy was mainly focused on grafting the LIMA to the left anterior descending and harvesting the RIMA as a free graft to construct a proximal Y anastomosis to graft the next largest target on the left system. A left RA or SVG was used to supplement the left system or the right coronary targets as an end‐to‐side graft from the mammary. For CPB, the cannulation sites can be in the distal ascending aorta, aortic arch, femoral artery, or axillary artery. If a safe disease‐free spot for the aortic cross‐clamp is available, then the ascending aorta is cross‐clamped and routine CABG is done with cold antegrade cardioplegia for myocardial protection. If the ascending aorta cannot be safely cross‐clamped, the surgical team is left to decide between doing FACAB or ARCAB. FACAB is done by cooling the systemic temperature to 28°C, fibrillating the heart by applying a shock using a fibrillatory device, then constructing all the distal anastomoses, and then performing the proximal anastomoses with the head down under low or short bouts of no flow in the aortic canula. If the ascending aorta has a large anterior atheroma that prevents proximal construction, then the systemic temperature is cooled down to 18°C and the ascending aorta is replaced under deep hypothermic circulatory arrest with a Dacron tube graft. The tube graft is then cross‐clamped and routine CABG is performed.

Perioperative outcomes were assessed by a trained database manager who was unaware of the goals of this study. Death was defined as death at any time during the index hospitalization. Myocardial infarction (MI) was defined as a fatal or nonfatal MI as evidenced by new Q waves on the electrocardiogram or by a peak in creatine kinase isoenzyme MB level greater than 50 IU/L that represented more than 7% of the total creatine kinase. Echocardiographic corroboration of a new MI was routinely sought but was not necessary for the diagnosis. For the purpose of this manuscript, stroke was defined as signs and symptoms of neurological deficit due to cerebrovascular causes that persist beyond 24 h or are interrupted by death within 24 h. Neurological deficits that resolved completely within 24 h were labeled as transient ischemic attacks (TIAs). Strokes were confirmed by a staff neurologist and CT scans. Renal insufficiency was defined as having a decrease in the calculated GFR to less than 60 ml/min. Completeness of revascularization was determined by successfully grafting all diseased coronary distribution territories. Renal failure was defined as the requirement for dialysis. Smoking was defined as a current history of smoking, or cessation within the 3 months preceding surgery. Hypertension, dyslipidemia, diabetes mellitus, and peripheral vascular disease are referred to medically or surgically treated conditions. As of the first quarter of 2020, the follow‐up was 98% complete. Crude survival was determined by matching the unique national identification number for each patient in the series with the national death registry. The cause of death was determined by reviewing the death certificate for those who matched in the registry. Patients who were still alive were directly contacted after obtaining permission at the time of the inquiry. Primary cardiac death was determined if the cause of death was acute coronary syndrome, cardiogenic shock, congestive heart failure, valvular heart disease, death during a cardiac procedure, or sudden cardiac death. History of stroke, TIA, and nonfatal MI were determined at the time of follow‐up inquiry by the study team.

Statistical analysis was performed using SPSS 21.0 software (SPSS Inc.). Categorical data were summarized as absolute numbers and percentages. Numeric data were summarized as the mean and standard deviation (SD) or median and interquartile range. The tested variables were grouped in 2 × *n* tables and the two‐group comparisons were made using the chi‐square test or Fisher exact test for categorical data. Continuous variables were tested using the Student *t*‐test or Mann–Whitney *U*‐test. The crude odds ratios (ORs) with 95% confidence intervals (CIs) were estimated using univariate logistic regression, and adjusted ORs with 95% CIs were estimated using multiple logistic regression analysis. Crude survival and event‐free survival curve comparisons were done using the log‐rank test. The results were expressed as hazard ratios (HRs), 95% CIs, and *p*‐values. A two‐tailed *p*‐value < 0.05 was considered significant for all statistical tests.

## 3 RESULTS

A total of 2100 consecutive patients were referred for isolated CABG within the study time frame (2004–2019). Of those CABG patients, 902 (45%) had CABG before the implementation of the protocol, and 1198 (57%) patients had CABG after implementation. A significantly larger number of patients had a screening with chest CT before surgery and EAU intraoperatively. This resulted in a significantly higher rate of detection of ascending aorta calcification and atheroma after the implementation of the protocol (Supporting Information: Accessory Table 1).

Furthermore, the chart review yielded 99 patients (4.7%) who were labeled as having SAA mandating re‐evaluation of the revascularization strategy. This cohort was divided based on the time of surgery into 30 patients in group C (30.3%) and 69 patients in group B (69.7%).

Demographic analysis (Table 1) revealed that patients in both groups were comparable and were predominantly older (>69 years) male patients who presented with a high prevalence of known risk factors for atherosclerotic disease: diabetes, hypertension, hyperlipidemia, and smoking. The B group had a higher percentage of patients with a past history of ischemic strokes or TIA (25% vs. 7%; *p* = 0.037). The B group also had more patients with chronic renal insufficiency (41% vs. 20%; *p* = 0.048) and more patients who presented with left main coronary artery stenosis (38% vs. 17%; *p* = 0.038). The surgical risk scores (EuroSCORE II) of the two groups were similar: 2.91% versus 2.34% in B and C, respectively (*p* = 0.670).

**Table 1:** Subject characteristics and comorbidities

Demographics	Controls (Group C)	“Brain‐before‐heart” (Group B)	
	*n* = 30	*n* = 69	*p* Value
Age (years) ± SD	69 ± 8	70 ± 6	0.420
Male sex	23 (78%)	52 (75%)	0.889
Diabetes	21 (70%)	54 (78%)	0.378
Hypertension	22 (73%)	59 (85%)	0.149
Hyperlipidemia	15 (50%)	45 (65%)	0.154
Smoking history	24 (80%)	57 (83%)	0.757
History of stroke/TIA	2 (7%)	17 (25%)	0.037
Carotid disease >70%	4 (13%)	18 (26%)	0.161
Peripheral vascular disease	6 (20%)	20 (28%)	0.350
NYHA Class III–IV	3 (10%)	12 (17%)	0.346
Left ventricular EF < 40%	4 (13%)	10 (15%)	0.879
Chronic renal insufficiency^a^	6 (20%)	28 (41%)	0.048
Chronic renal replacement	2 (7%)	6 (9%)	0.734
Left main CAD	5 (17%)	26 (38%)	0.038
Median EuroSCORE II (without aorta)	2.34% (1.75, 4.40)^b^	2.91 (1.5, 6.44)^b^	0.670

Abbreviations: CAD, coronary artery disease; EF, ejeciton fraction; NYHA, new york heart association; TIA, transient ischemic attack.

^a^ Calculated glomerular filtration rate <60 ml/min.

^b^ 25th and 75th percentiles.

The analysis of how the stroke prevention protocol impacted the use of the six revascularization strategies used in SAA (Table 2) showed that the techniques were used in the C group in the following order of preference: OPCAB (40%), ARCAB (37%), hybrid (7%), PCI (6%), FCAB (7%), and CABG (3%). In comparison, the order of preference of techniques used in the B group was the following: OPCAB (35%), PCI (26%), hybrid (13%), ARCAB (12%), CABG (11%), and FCAB (3%). Of the total of 36 patients in the cohort who had OPCAB, 21 (58%) underwent an aorta “no touch” OPCAB. Only 2 patients were done before the implementation of the new stroke prevention strategy and 19 were done after its implementation. There was a clear tendency to increased the use of PCI after adopting the new protocol in the B group (26% vs. 7%; *p* = 0.027). Also, the use of ARCAB in the B group declined significantly with the adoption of the protocol (12% vs. 37%; *p* = 0.004) (Supporting Information: Accessory Figure 1).

**Table 2:** The impact of the stroke prevention protocol on the use of the six revascularization strategies used in SAA

	Controls (Group C)	“Brain‐before‐heart” (Group B)	
Technique	*n* = 30	*n* = 69	*p* Value
Percutaneous coronary intervention (PCI)	2 (6%)	18 (26%)	0.027
On‐pump with cross‐clamp (CABG)	1 (3%)	8 (11%)	0.270
Off‐pump coronary artery bypass (OPCAB)	12 (40%)	24 (35%)	0.620
Hybrid revasculariztion (OPCAB + PCI)	2 (7%)	9 (13%)	0.496
Aortic replacement coronary artery bypass (ARCAB)	11 (37%)	8 (12%)	0.004
Fibrillating coronary artery bypass (FACAB)	2 (7%)	2 (3%)	0.382

Abbreviations: CABG, coronary artery bypass grafting; SAA, severe atherosclerosis of the ascending aorta.

The patients who had surgical revascularization (20 patients were excluded from this outcome as they had PCI) in the B group had more multiple arterial grafts (38% vs. 13%), and more complete revascularization (96% vs. 79%; *p* = 0.021). Perioperative stroke and TIA were more pronounced in the C group when compared with the B group (18% vs. 2%, respectively; *p* = 0.019). With the exception of three patients in the C group who required dialysis for acute kidney injury, all other immediate surgical outcomes were comparable (Table 3). Revascularizing the SAA before the initiation of the stroke prevention protocol was significantly associated with a perioperative embolic neurological insult (HR: 1.87, 95% CI: 1.2–9.8, *p* = 0.034). This association remained significant after adjusting for age, history of stroke, carotid stenosis, peripheral vascular disease, low ejection fraction (ejeciton fraction < 40%), and left main coronary disease (HR 1.80, 95% CI: 1.4–2.21, *p* = 0.023).

**Table 3:** In‐hospital surgical outcomes: Comparison of cases with controls

	Controls (Group C)	Brain‐before‐heart (Group B)	
	*n* = 28	*n* = 51	*p* Value
Median number of grafted targets	3	3	0.270
Use of multiple arterial grafts	4 (13%)	26 (38%)	0.001
Completeness of revascularization	24 (79%)	49 (96%)	0.021
Stroke/TIA	5 (18%)	1 (2%)	0.019
Postoperative MI	2 (7%)	3 (6%)	0.826
Bleeding requiring re‐exploration	4 (14%)	2 (4%)	0.178
Acute kidney injury requiring dialysis	3 (11%)	0 (0%)	0.041
Operative death	2 (7%)	3 (6%)	1.000

Abbreviations: MI, myocardial infarction; SAA, severe atherosclerosis of the ascending aorta.

**Figure 2:**
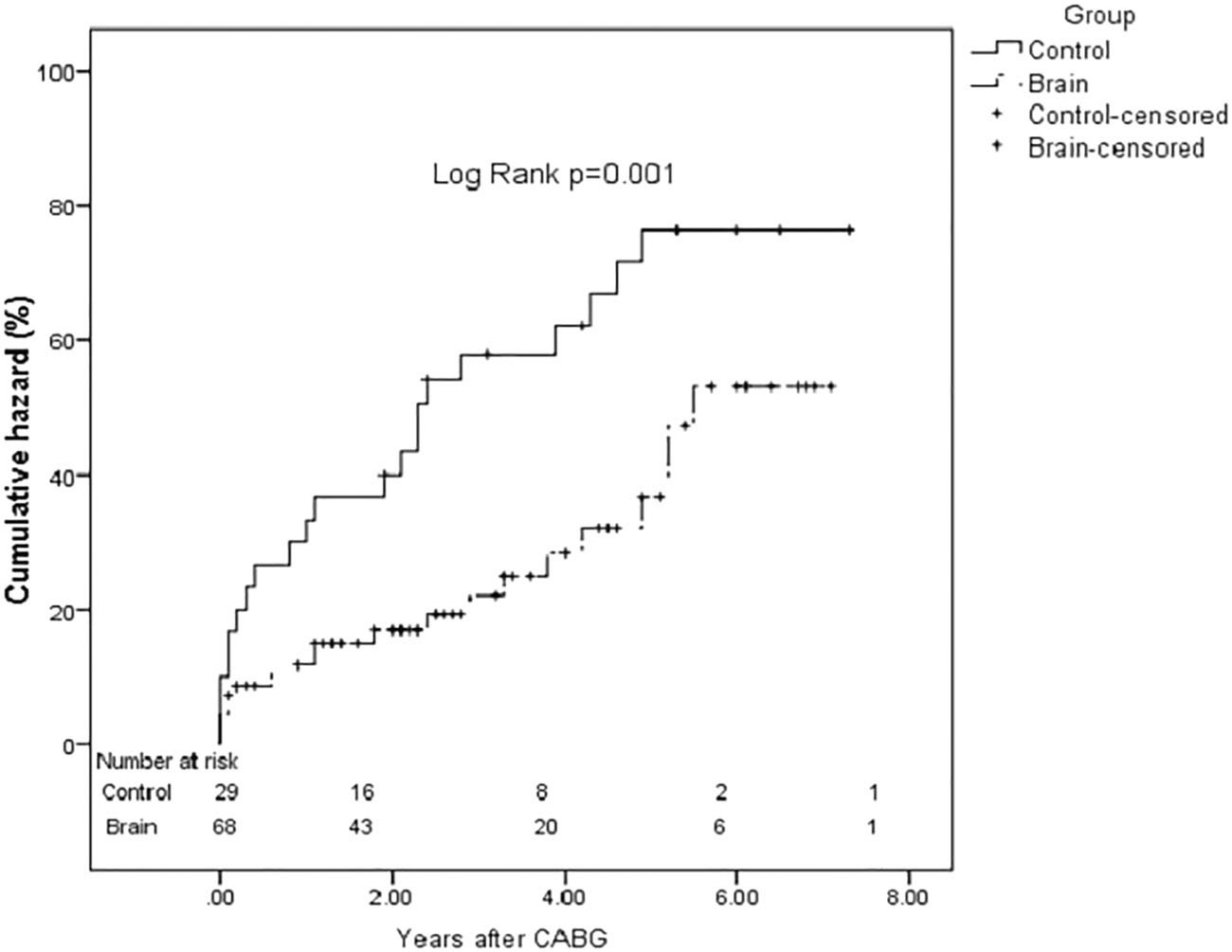
The cumulative hazard risk cases and controls up to 8 years post‐CABG. CABG, coronary artery bypass grafting.

Crude survival at the end of 8 years (median follow‐up 2.3 years) from the time of revascularization was not statistically significantly different between groups B and C (60.7% vs. 43.4%, respectively; *p* = 0.102) (Supporting Information: Accessory Figure 2). The cumulative hazard risk for the composite endpoints of cardiac death, nonfatal MI, and stroke/TIA over the 8 years since revascularization was significantly higher in the C group (76.3% vs. 53.1; *p* = 0.001) (Figure 2) (Supporting Information: Accessory Table 2). As for the 20 patients who had PCI instead of the high‐risk surgical revascularization, the outcomes in the 8 years after intervention were: none had stroke/TIA, 30% died due to cardiac causes, and 10% had nonfatal MI.

## 4 DISCUSSION

Increased awareness and early recognition of ascending aortic atherosclerosis are perhaps the most crucial measures in reducing the devastating cerebro‐embolic complications associated with surgical revascularization. For this subset of patients with SAA, minimizing cerebral embolization during the manipulation of an atherosclerotic ascending aorta becomes a pressing priority over achieving complete myocardial revascularization. In essence, this is the logic behind the brain‐before‐heart strategy, as the spectrum of therapeutic options for ischemic heart disease after incomplete revascularization is wider than the spectrum of therapies available for postoperative stroke.^5^ The implementation of a preemptive strategy to detect and modify the revascularization methods over the second half of the study timeline has yielded a greater number of detected cases of SAA and included more patients with a history of stroke, chronic renal dysfunction, and patients with left main coronary stenosis. More importantly, the rate of perioperative strokes under the new protocol was significantly reduced.

One big advantage of the preemptive approach in SAA is the ability to reduce the element of undesirable surprises during surgery. As with all our B group subjects, Park et al.^10^ found that half of the patients who were subjected to routine CT angiography before CABG (in our study it was plain chest CT) had considerable changes in their surgical evaluations, surgical decision‐making, and follow‐up plans. The authors recommend its routine use for evaluation before CABG in older than 60‐year‐old patients unless contraindicated. Intraoperative evaluation with EAU adds further precision to the surgical technique and surgical decision‐making. In a prospective study of 55 patients aged 70 and above who were randomized to either EAU or standard digital palpation during routine CABG, it found that EAU led to modifications in the intraoperative surgical management in one‐third of patients undergoing CABG.^11^ Yet, this did not translate to a difference in clinical outcomes, including the number of embolisms detectable via trans‐cranial Doppler. Another and larger retrospective study by Luthra et al. found that intraoperative EAU clearly helped in reducing the incidence of neurological events, especially in high‐risk patients aged 70 and above.^12^ Our own results show that both preoperative CT screening and intra‐operative EAU play a key role in operative decision making and can sway the decision sometimes in favor of not proceeding with surgical revascularization and performing PCI instead. Moreover, the –almost‐ universal use of intraoperative EAU in the later part of the study has made it possible to detect “soft” astheromas that would otherwise go undetected by CT scan or digital palpation. This –in part‐ might also explain the significant reduction of perioperative stroke rates in the intervention group. This was the case for 20 patients in the series, most of whom (18 out of 20) were in the B group. In both groups, the procedure of choice was OPCAB, which came as no surprise as it has been well known for over two decades that it has the least risk of stroke, especially the “aortic no touch” technique.^7^, ^13^ With the application of the protocol, some notable differences were noted in the use of some of the techniques: ARCAB fell out of favor significantly, from 37% to 12% (*p* = 0.004); significantly more PCIs were done in the B group; more hybrid revascularization was done in the B group; and surgeons became more comfortable performing CABG with cross‐clamping after mapping the ascending aorta with EAU. The use of FCAB remained low for both groups.^9^

All the surgical techniques used in this study have scientific merit in mitigating the effects of ischemic stroke and poor survival in SAA, but it is the application of our new protocol and algorithm that we tested against the operative outcomes and event‐free survival. We know of no study conducted on patients with SAA that is comparable to the current study. Our operative results clearly show that, since the application of the new protocol, we observed better use of arterial grafts (2 instead of 1), higher complete revascularization (96% vs. 79%), and more importantly, significantly fewer perioperative strokes/TIAs (2% vs. 18%), even after adjusting for other risk factors associated with stroke and cerebrovascular disease. The clinical endpoints of cardiac death, nonfatal MI, and stroke/TIA for the median follow‐up of 2.3 years clearly favor the outcomes in the patients who had revascularization under the new protocol in our cohort. In a long‐term follow‐up study on 1957 patients over 50 who underwent cardiac surgery, Davila‐Roman et al. found that over the period of 7 years after surgery, the patients who had “severe” atherosclerosis of the ascending aorta based on EAU criteria had a greater than 3‐fold increase in the incidence of both neurologic events and mortality compared with those with less severe atherosclerotic disease.^3^ Our findings show that with proper selection and individualized decision‐making in such high‐risk patients, perioperative and mid‐term outcomes can improve.

This study is unique in the way it analyzed the immediate and mid‐term impacts of applying a stroke prevention protocol for detecting and managing patients with SAA. The revascularization strategy was individualized and based on an algorithm that uses pre‐ and intra‐operative imaging modalities. Despite this, our findings are limited by a number of factors. The study groups were managed and treated in different time periods. Although the time gap is not big, nonmeasurable subtleties such as an improved learning curve, better equipment, and access to more sensitive imaging technologies can account for better surgical results and outcomes. No distinction was made between the “soft” and calcific atheromas in the ascending aorta as the earlier can only be detected by EAU and has a higher propensity to cause stroke. The study's retrospective nature and single institution experience are inherent limitations. Determining the cause of death solely from death certificates can be inaccurate at times. Reporting nonfatal events based on verbal communication is subjected to recall bias and inaccurate reporting. The late outcomes of PCI as an alternative to surgical revascularization in SAA were not specifically analyzed in this study, due to the small sample size.

In conclusion, this study is an attempt to evaluate practical measures for coping with SAA in the increasing number of patients who need surgical revascularization. Early detection and individualized therapeutic strategies play an important role in reducing the devastating neurologic insults that can result from manipulating a diseased ascending aorta during surgery.

## AUTHOR CONTRIBUTIONS

Rakan I. Nazer conceived the study, helped plan the methodology, helped collect the data, and wrote the manuscript. Ali M. Albarrati helped plan the methodology, helped collect the data, did the analysis, and reviewed the manuscript.

## CONFLICT OF INTEREST

The authors declare no conflict of interest.

## Supporting information

Supporting information.

Supporting information.

Supporting information.

Supporting information.

## Data Availability

The data sets used and analyzed during the current study are available from the corresponding author on reasonable request.
