# Supplementary material for: The brain‐before‐heart strategy for coronary artery bypass grafting in the severely atherosclerotic aorta: A single‐institution experience
Source: Clin Cardiol. 2022 Sep 19;45(12):1264–71. doi: 10.1002/clc.23913 (PMC9748750; doi:10.1002/clc.23913)

**Accessory figure 1**: Alternative revascularization strategies in sever atherosclerotic aorta


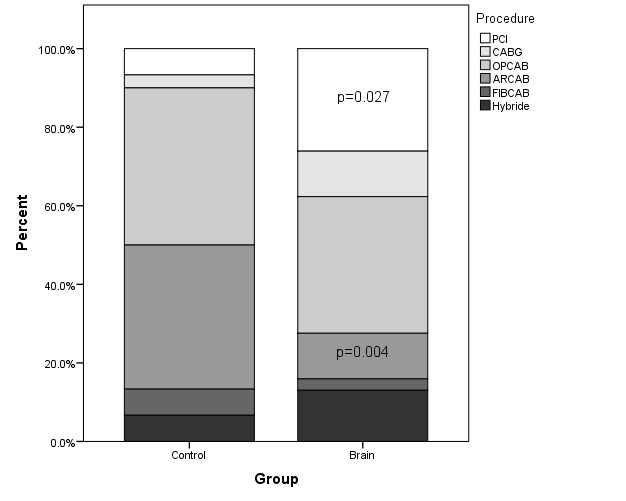

Supplement: Supplementary file 1 — Supporting information. [file CLC-45-1264-s002.docx]
