# Supplementary figures and images for: The brain‐before‐heart strategy for coronary artery bypass grafting in the severely atherosclerotic aorta: A single‐institution experience
Source: Clin Cardiol. 2022 Sep 19;45(12):1264–71. doi: 10.1002/clc.23913 (PMC9748750; doi:10.1002/clc.23913)

**Accessory figure 2:** Crude survival rates of cases and controls up to 8 years post CABG


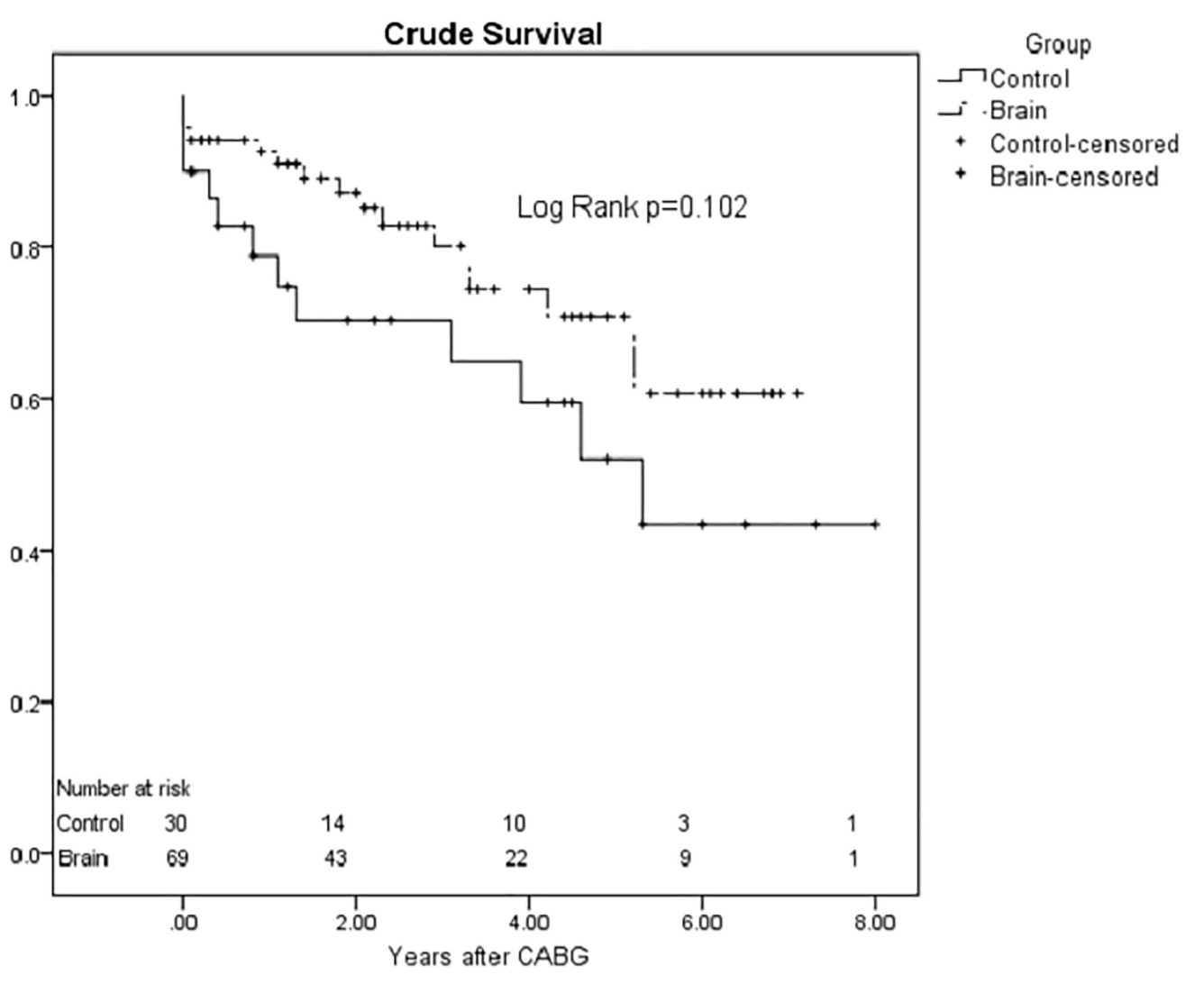

Supplement: Supplementary file 2 — Supporting information. [file CLC-45-1264-s001.docx]
