# Supplementary material for: The brain‐before‐heart strategy for coronary artery bypass grafting in the severely atherosclerotic aorta: A single‐institution experience
Source: Clin Cardiol. 2022 Sep 19;45(12):1264–71. doi: 10.1002/clc.23913 (PMC9748750; doi:10.1002/clc.23913)
Supplement: Supplementary file 3 — Supporting information. [file CLC-45-1264-s003.docx]

**Accessory table 1**: Screening for atheromas in the ascending aorta.

|  | **Controls** | **"Brain-before-heart"** | ***p*-value** |
| --- | --- | --- | --- |
|  | (Group C) | (Group B) |  |
|  | n = 902 | n = 1198 |  |
| Screening modality |  |  |  |
|  |  |  |  |
| Chest CT screening | 550 (61%) | 1119 (93.4%) | <0.001 |
| Chest CT positive for Ca+ | 36 (4%) | 74 (6.2%) | 0.029 |
| Epiaortic US (EUA) | 195 (21.6%) | 1180 (98.5%) | <0.001 |
| Epiaortic US (EUA) positive for atheroma | 30 (3.3%) | 68 (5.7%) | 0.012 |
