# Supplementary material for: The brain‐before‐heart strategy for coronary artery bypass grafting in the severely atherosclerotic aorta: A single‐institution experience
Source: Clin Cardiol. 2022 Sep 19;45(12):1264–71. doi: 10.1002/clc.23913 (PMC9748750; doi:10.1002/clc.23913)
Supplement: Supplementary file 4 — Supporting information. [file CLC-45-1264-s004.docx]

**Accessory table 2:** Follow-up end points

|  | (Group C) n = 30 | (Group B) n = 69 |
| --- | --- | --- |
| Cardiac death | 7 (23%) | 6 (9%) |
| None fatal MI | 6 (20%) | 4 (6%) |
| Stroke/ TIA | 6 (20%) | 10 (14%) |
